# Effect of intermittent aerobic exercise on sleep quality and sleep disturbances in patients with rheumatoid arthritis – design of a randomized controlled trial

**DOI:** 10.1186/1471-2474-15-49

**Published:** 2014-02-21

**Authors:** Katrine Løppenthin, Bente Appel Esbensen, Poul Jennum, Mikkel Østergaard, Jesper Frank Christensen, Tanja Thomsen, Julie Schjerbech Bech, Julie Midtgaard

**Affiliations:** 1 Research Unit of Nursing and Health Science, Glostrup Hospital, Copenhagen University, Copenhagen, Denmark; 2 Center for Rheumatology and Spine Diseases, Glostrup Hospital, Copenhagen University, Copenhagen, Denmark; 3 Department of Public Health, Faculty of Health and Medical Sciences, University of Copenhagen, Copenhagen, Denmark; 4 Danish Centre for Sleep Medicine, Department of Clinical Neurophysiology, Glostrup Hospital, Copenhagen University, Copenhagen, Denmark; 5 Department of Clinical Medicine, Faculty of Health and Medical Sciences, University of Copenhagen, Copenhagen, Denmark; 6 University Hospitals Centre for Health Care Research, Copenhagen University Hospital, Copenhagen, Denmark; 7 Department of Rheumatology, Frederiksberg Hospital, Copenhagen University, Copenhagen, Denmark

**Keywords:** Sleep quality, Sleep disturbances, Fatigue, Polysomnography, Cardiorespiratory fitness, Intermittent training, Rheumatoid arthritis

## Abstract

**Background:**

Poor sleep is prevalent in patients with systemic inflammatory disorders, including rheumatoid arthritis, and, in addition to fatigue, pain, depression and inflammation, is associated with an increased risk of co-morbidity and all-cause mortality. Whereas non-pharmacological interventions in patients with rheumatoid arthritis have been shown to reduce pain and fatigue, no randomized controlled trials have examined the effect of non-pharmacological interventions on improvement of sleep in patients with rheumatoid arthritis. The aim of this trial was to evaluate the efficacy of an intermittent aerobic exercise intervention on sleep, assessed both objectively and subjectively in patients with rheumatoid arthritis.

**Methods/design:**

A randomized controlled trial including 44 patients with rheumatoid arthritis randomly assigned to an exercise training intervention or to a control group. The intervention consists of 18 session intermittent aerobic exercise training on a bicycle ergometer three times a week. Patients are evaluated according to objective changes in sleep as measured by polysomnography (primary outcome). Secondary outcomes include changes in subjective sleep quality and sleep disturbances, fatigue, pain, depressive symptoms, physical function, health-related quality of life and cardiorespiratory fitness.

**Discussion:**

This trial will provide evidence of the effect of intermittent aerobic exercise on the improvement of sleep in patients with rheumatoid arthritis, which is considered important in promotion of health and well-being. As such, the trial meets a currently unmet need for the provision of non-pharmacological treatment initiatives of poor sleep in patients with rheumatoid arthritis.

**Trial registration:**

ClinicalTrials.gov Identifier: NCT01966835

## Background

Restorative sleep has an important role in maintaining health [1], with disrupted or lower levels of sleep being related to serious outcomes such as increased risk of morbidities [2,3] and ultimately all-cause mortality [4]. Sleep disturbances and poor sleep quality are prevalent complaints in patients with chronic diseases [5] including rheumatoid arthritis (RA). RA is a chronic inflammatory disease associated with increased mortality, high morbidity and reduced health related quality of life [6,7]. It affects between 0.1 and 0.5% of adults in developed countries and is three times more frequent in women than men. In addition to pain and fatigue, poor sleep has been identified as a major concern by patients with RA [8] affecting 50-70% of the patients [9]. Importantly, poor sleep in RA may be affected by pain or may contribute to increased pain and fatigue [10-13], and is associated with depression [14,15] and inflammation [16]. Thus, addressing poor sleep quality may be important in promoting health and well-being in patients with RA.

Few studies have examined treatments for managing poor sleep in patients with RA. Those carried out have examined sleep medication (eszopiclone, zopiclone, valerian) [17-19] or biological therapies (tocilizumab, infliximab, anti-tumor necrosis factor α, abatacept) [20-23]. However, non-pharmacological psychological interventions (e.g. cognitive behavioral therapy) [24] have been shown to be an effective alternative for patients with sleep disturbances and may have a longer-lasting effect, with no side effects. However, psychological interventions (e.g. cognitive behavioral therapy) typically require highly skilled clinicians and may therefore be expensive and time-intensive to deliver [24]. Also, while psychological interventions are promising, they mainly affect mental health and/or include low-intensity behavioral interventions (e.g. relaxation) with little or no effect on functional and physiological outcomes.

Cross-sectional studies have shown that physical inactivity increases the likelihood of reporting poor sleep, even after controlling for age, depression and pain [25,26]. Furthermore, it has been reported that, independent of age and sex [27], maximal aerobic capacity is lower in patients with insomnia compared to those without it. In addition, physical exercise interventions have been shown to be a feasible and effective non-pharmacological treatment modality for improving sleep in healthy and in clinical populations [28-31]. Exercise interventions is known to improve cardiorespiratory fitness, muscle strength and functional ability [32], as well as health-related quality of life [33], and to reduce co-morbidity [34] in patients with RA. Thus, physical exercise interventions may be a viable non-pharmacological alternative for both prevention and treatment of poor sleep in addition to the simultaneous benefits for numerous health parameters in the human body.

The effect of physical exercise interventions on poor sleep has not hitherto been examined in patients with RA. However, a systematic review including older adults has shown that exercise of moderate intensity has a moderately positive effect on improvement of self-reported sleep [31]. In addition, a randomized controlled trial (RCT) [35] of adults > 55 years old found that moderate intensity exercise decreased objectively measured Stage 1 sleep and number of nighttime awakenings, and increased Stage 2 sleep. However, high-intensity aerobic exercise may also be assumed to be efficient for improving sleep. For patients who do not possess the necessary fitness level to perform continuous high-intensity exercise, aerobic intermittent training (defined as: vigorous exercise performed at a high intensity for a brief period of time interposed with recovery intervals at low to moderate intensity [36]), may provide an alternative to continuous high-intensity exercise.

In summary, the significant morbidity associated with poor sleep and the multifactorial impact of physical exercise suggest that an intermittent aerobic exercise training intervention may constitute a promising non-pharmacological treatment approach for the management of poor sleep in patients with RA.

Against this background, the aim of this trial is to assess the efficacy of an aerobic intermittent exercise training intervention on sleep quality and sleep disturbances in patients with RA. The primary hypothesis is that intermittent aerobic exercise will be superior to no exercise in the improvement of objectively measured sleep. The secondary hypotheses are that patients randomly assigned to the exercise intervention will show greater improvement in subjective sleep quality and sleep disturbances, cardiorespiratory fitness, and physical function as well as reduced pain, fatigue and depressive symptoms and improved health-related quality of life compared to patients randomly assigned to the no-intervention (control group).

## Methods

### Design

**Figure 1:**
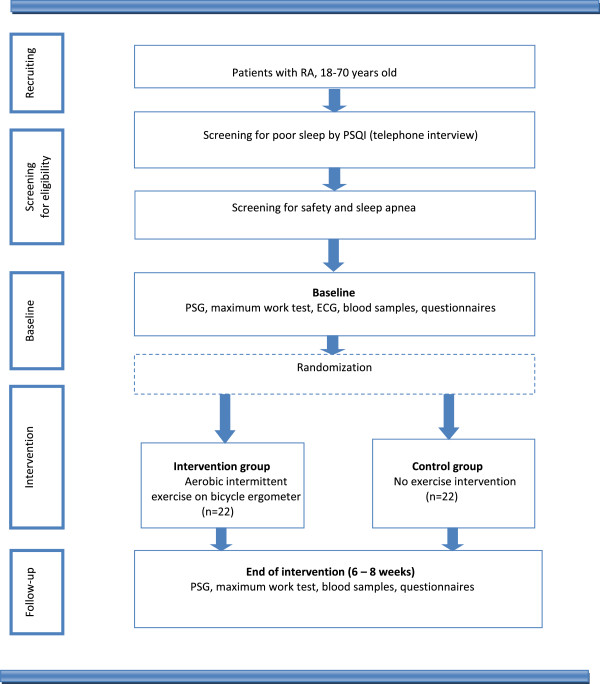
Flow of patients through the randomized controlled trial.

The present RCT is single blinded, with an aerobic intermittent exercise training intervention and a primary endpoint of sleep measured by polysomnography (PSG). Flow of patients through the trial is shown in Figure 1.

#### Sample size

The sample size calculation is based on the sleep component `sleep efficiency´. A previous RCT [37], using PSG as measurement of sleep, examined the effect of a non-pharmacological intervention (cognitive behavioral therapy) on sleep disturbances in patients with insomnia and found a mean sleep efficiency of 81.4% (SD 7.4). The normal value for sleep efficiency is >85%. Thus the aim for the patients in the exercise intervention was to achieve a sleep efficiency of at least 86%. On this basis, and with a significance level of α = 5% and a power of 80%, it is estimated that 36 patients should take part in this trial. A drop-out rate of 20% is expected, which means that 44 patients are included, of which 22 are randomly assigned to the intervention group and 22 to the control group.

#### Population

The trial consists of 44 patients with a clinical diagnosis of RA based on The American College of Rheumatology (ACR)/The European League Against Rheumatism (EULAR) criteria.

#### Eligibility criteria

### Inclusion criteria

Patients are eligible to participate if they are aged 18-70; have a diagnosis of RA; report poor sleep quality (Pittsburgh Sleep Quality Index (PSQI) global score >5), have low disease activity score on 28 joints (DAS28) <3.2, have a normal 12-lead Electrocardiogram (ECG) and understand Danish.

### Exclusion criteria

Patients are excluded if they have documented sleep apnea (Apnea-Hypopnea Index (AHI) >15/hour); have night work during the period in which the intervention takes place; are pregnant or are breast-feeding, are being treated with steroids, hypnotics, antidepressants or antipsychotics, have cardiac symptoms corresponding to New York Heart Association (NYHA) Functional Classification >2; are regularly physically active (self-reported aerobic exercise >3 times per week).

#### Trial setting and recruitment

The patients are recruited from the Rheumatology outpatient clinics at two Copenhagen University Hospitals, Glostrup and Frederiksberg, in the Capital Region of Denmark. Potentially eligible patients receive written information by mail or are contacted in the outpatient clinics at Glostrup and Frederiksberg Hospital and receive a telephone call a few days later. If the patients are interested in participating in the trial, a telephone screening is conducted using the PSQI to clarify whether they fulfill the criteria for poor sleep (PSQI global score >5).

#### Screening

Eligible patients are screened for sleep apnea. This screening is performed by cardio-respiratory monitoring (CRM) consisting of electromyography of the tibialis anterior muscles, and electrocardiogram including channels of digital oximetry, nasal pressure and respiratory movements (Xtrace thoracic and abdominal straps). Patients with moderate to severe sleep apnea, AHI >15/hour, are excluded from the trial, and are referred to the Center for Sleep Medicine for treatment for sleep apnea.

Safety: During the screening period, to ensure that their health permits high-intensity intermittent aerobic training, the patients are asked, by means of a structured screening instrument, about their heart and lung function to ensure that they have no symptoms and no limitations on ordinary physical activity, e.g. shortness of breath when walking, climbing stairs etc. or mild shortness of breath and/or angina. Furthermore, the patients will have had an ECG taken to ensure the safety of participating in the aerobic intermittent training program.

#### Randomization and blinding

Included patients are randomly assigned to one of the two following conditions: aerobic intermittent training (n = 22) or control group (n = 22). Randomization is performed by computer-generated `random numbers´ with a 1:1 allocation ratio. Evaluation of primary outcome is performed by experienced polysomnographic technicians who are blinded to group allocation.

#### Intervention

The intervention consists of a total of 18 aerobic intermittent training sessions (20-30 minutes/session) spread over a maximum of eight weeks (2-3 times/week) as shown in Table 1. The training sessions are performed on bicycle ergometers (Kettler) and supervised by trained physiotherapists. Each session is initiated by a 5-minute warm-up period, followed by 4 blocks of 5-7.5 minutes alternating periods of continuous moderate intensity (40-50% watt max) and periods of intermittent high-intensity aerobic exercise (70-80% watt max) [38] and finally a 5-minute cool-down period. Exercise workload (watt) is determined individually by a watt max test at baseline, and calculated from the maximum load reached adjusted for the number of seconds performed at the highest load. The goal of the intervention is for each patient to attend at least 14 out of the 18 planned exercise sessions.

**Table 1:** Exercise training program for patients randomized to 18 sessions of aerobic intermittent training

**18 sessions of aerobic intermittent training (6 – 8 weeks duration)**					
**Session 1 – 6**			**Session 7 - 18**		
Duration	20 minutes aerobic intermittent training including 5 minute warm-up and cool-down		Duration	30 minutes aerobic intermittent training including 5 minute warm-up and cool-down	
Bock 1 (5 min)	Continuous exercise	55-60% Wmax (70-80% HR)	Block 1 (7.5 min)	Continuous exercise	55-60% Wmax (70-80% HR)
Block 2 (5 min)	Intermittent training 3 x 60 -30 sec.	70-80% Wmax (80-90% HR)	Block 2 (7.5 min)	Intermittent training 4 x 60 -30 sec.	70-80% Wmax (80-90% HR)
Block 3 (5 min)	Intermittent training 3 x 60-30 sec.	70-80% Wmax (80-90% HR)	Block 3 (7.5 min)	Intermittent training 5 x 60 - 30 sec.	70-80% Wmax (80-90% HR)
Block 4 (5 min)	Continuous exercise	55-60% Wmax (70-80% HR)	Block 4 (7.5 min)	Continuous exercise	55-60% Wmax (70-80% HR)

Wmax = watt max, HR = Heart rate.

### Monitoring

For each exercise session any changes in the prescribed exercise intensity (watt) is noted. Patients’ heart rate (Polar) is monitored and noted for each training block, and the overall mean and maximum heart rate are registered after each session. In addition, the patients’ rated perceived exertion is recorded using the Borg Scale (6-20 rating).

#### Control group

The control group receives no exercise intervention during the test period and is encouraged to maintain a normal everyday life.

#### Trial endpoints

##### Data collection

Objective physiological as well as patient reported outcomes are collected at baseline and at the end of the intervention (6- 8 weeks).

### Assessment of primary endpoint

The primary outcome of the trial is changes in sleep as measured objectively by PSG at baseline and at the end of the intervention. PSG measurements are performed at the patient’s home in order for them to sleep in familiar surroundings. However, the primary investigator performs electrode placement and tests the equipment at Glostrup Hospital. Sleep monitoring is performed using Trackit™ Ambulatory EEG (Lifelines Ltd, 7 Clarendon Court, Over Wallop, Nr. Stockbridge, Hants, UK). The evaluation of the PSG measurements is performed following the guidelines of the American Academy of Sleep Medicine (AASM standard) 2007 [39].

### Assessment of secondary endpoints

#### Objective physiological measures

##### Cardiorespiratory fitness

The patients’ cardiorespiratory fitness is assessed by an incremental maximum work test on a bicycle ergometer as described by Andersen et al [40]. The test is modified to accommodate the patients’ fitness level in order to obtain a test period of 5 minutes or more. Following a 5-minute warm-up period on individual steady state work intensity the work load increases by 20 watt each minute until exhaustion is reached. Maximum work load (Watt), maximum heart rate (beats per minute) and time to exhaustion are recorded as test results. This test has been shown in previous studies to be suitable when estimating cardiorespiratory fitness in patients with chronic disease [41].

#### Plasma

C-reactive protein (CRP) and hemoglobin are assessed at baseline and at the end of the intervention. If Anti-CCP and IgM rheumatoid factor are not available these blood samples will also be taken. In addition, blood pressure, weight and height will be measured.

#### Disease activity

Disease Activity Score (DAS28) is calculated from the number of swollen and tender joints (28 joints), CRP and the patients’ global assessment of how much arthritis affects their lives.

##### Patient reported outcomes

The trial includes six standardized validated questionnaires assessing changes in patient-reported outcomes.

#### Sleep quality and sleep disturbances

The Pittsburgh Sleep Quality Index (PSQI) [42] measures self-reported sleep quality and disturbances during the previous four weeks. PSQI has 19 items and measures 7 components of sleep: subjective sleep quality, sleep latency, sleep duration, sleep disturbance, use of sleeping medication, habitual sleep efficiency and daytime dysfunction.

#### Sleepiness

The Epworth Sleepiness Scale (ESS) [43] is a self-reported questionnaire that measures daytime sleepiness. The questionnaire consists of 8 items with a respondents format 0 = would never doze, 1 = slight chance of dozing, 2 = moderate chance of dozing, and 3 = high chance of dozing.

#### Sleep pattern

Patients complete sleep diaries for 14 days after each PSG measurement in order to monitor and describe potential changes in their sleep. The sleep diary contains self-reported information about what time they go to bed and get up, as well as the number of awakenings during the night and daytime dysfunction.

#### Fatigue

The Bristol Rheumatoid Arthritis Fatigue Multi-Dimensional Questionnaire (BRAF MDQ) [44] consists of 20 items including four sub-scale scores of physical fatigue, living with fatigue, cognitive fatigue and emotional fatigue. The questionnaire consists of four options; “not at all”, “a little”, “quite a bit”, “very much”.

#### Depression

The Center for Epidemiological Studies-Depression (CES-D) [45] consists of 20 items evaluating depressive symptomatology and attitudes. The patients indicate how often, over the previous week, they had experienced each of the 20 symptoms on a scale ranging from 0 to 3: rarely or none of the time; some or a little of the time; occasionally or a moderate amount of the time; most or all of the time.

#### Physical function

The Health Assessment Questionnaire (HAQ) [46], includes 20 items to assess activity limitation in 8 dimensions of activities of daily living: dressing and grooming, arising, eating, walking, hygiene, reach, grip, and common daily activities. In addition, in the HAQ questionnaire there is also a Visual Analogue Scale (VAS) for pain, fatigue and patient global assessment indicating how much arthritis affects their lives.

#### Health-related quality of life

EuroQol (EQ-5D-5 L) [47] measures health-related quality of life. The questionnaire contains five dimensions: mobility, self-care, usual activities, pain/discomfort and depression/anxiety. Each dimension has five levels: ‘no problems’, ‘slight problems’, ‘moderate problems’, ‘severe problems’ and ‘extreme problems’.

#### Health status and behavior

Information about self-reported co-morbidity, age, gender, education, work, income, physical activity, smoking, alcohol and caffeinated drink consumption is recorded. Patients are also asked for information about current medical treatment.

### Statistical analyses

Continuous variables, i.e., sleep, cardiorespiratory fitness, physical function, pain, fatigue, depressive symptoms and health-related quality of life will be presented as either means with corresponding 95% confidence limits or as medians and interquartile range (IQR). The primary and secondary endpoints will be reported as a two-sample t-test comparing change scores in the two randomized groups. The primary outcome variables in the regression model will be sleep parameters. The regression model will allow control for the baseline value of the trial endpoints and other confounding variables that may impact changes in sleep parameters (e.g. age and gender). Categorical data, i.e., single questionnaire items, will be reported as proportions and compared across randomized groups using chi-squared tests or logistic regression. Significance level will be set at 0.05. All tests will be two-tailed. The primary analysis will compare changes in the intervention vs. the control group using the intention-to-treat principle including all available data. Missing observations will be handled according to the missing at random assumption. The secondary analysis will include only patients attending at least 14 exercise sessions (80%) out of the prescribed 18 (i.e. per protocol analysis). Finally, correlations will be performed to examine the dose-response relation between changes in the primary outcome (sleep) and cardiorespiratory fitness. Data entry will be undertaken using a secure web server and statistical analysis will be performed using Statistical Analysis Systems (SAS) version 9.3.

### Qualitative investigation and methods

Qualitative, semistructured interviews will be held with purposefully selected sample of patients from the intervention group. These interviews will highlight patients’ experience with intermittent aerobic exercise including potential changes in pain, fatigue and sleep.

### Ethical considerations and confidentiality

The trial follows international ethical guidelines of informed consent in clinical trials. Patients receive written and verbal information about the trial to ensure they are able to give informed consent. Participation is voluntary and patients can withdraw their consent at any time during the trial with no consequences for their treatment. Research ethics approval to conduct this trial was obtained though the Regional Ethics Committee (Journal No. H-1-2012-151) and the trial has been registered in http://www.clinicaltrials.gov (NCT01966835). The trial is approved by the Danish Data Protection Agency (Ref. No. 711-1-08). All information collected during the course of the trial is kept confidential in accordance with Danish Data Protection Agency rules.

## Discussion

The present trial will contribute with empirical evidence to the field of exercise and sleep that is currently characterized by premature empirical results but with significant potential as a non-pharmacological approach for improving sleep and with several aspects beneficial to health. The mechanisms of how physical exercise may reduce poor sleep are yet unknown. One hypothesis may be that sleep decrease symptoms of depression [48], another is that exercise engages with the two-process model of circadian and homeostatic regulation [49] or stimulates the restorative function during sleep and triggers an increase in body temperature [50,51]. The post-exercise drop in temperature may promote sleep onset latency and slow-wave sleep.

The present trial is superior to others within the field of exercise and sleep in three ways. Firstly, we have chosen the golden standard method for measuring sleep (i.e. PSG). In comparison, the majority of previous studies with the aim of improving sleep through physical exercise interventions have relied on self-reported instruments such as PSQI and only one trial has used polysomnographic assessment to measure sleep objectively [35].

Secondly, whereas current exercise trials typically apply continuous moderate intensity aerobic exercise training, we have chosen to apply aerobic intermittent training. While regular continuous aerobic exercise is known to improve a wide range of health outcomes, including cardiorespiratory fitness in both healthy people and clinical populations, aerobic intermittent training of shorter duration may result in an equal or superior improvement in fitness [36].

Finally, this is the first trial within the field of RA to evaluate the effect of aerobic intermittent training on improvement in sleep quality and sleep disturbances. The detailed information collected in this trial regarding the exact completed adherence to the exercise regime (including intensity (watt) as well as pulse monitoring during each training block and interval, and objective measured sleep duration, sleep stages, and sleep quality) is expected to provide important experimental results that can contribute with evidence of both aerobic intermittent training and poor sleep, including causal interactions between the two.

### Trial status

At the time of manuscript submission the patients are being recruited to the trial.

## Competing interests

The authors declare that they have no competing interest.

## Authors’ contributions

The authors KL, BAE, PJ, MØ, JFC and JM participated in the planning and design of the trial. KL has drafted the manuscript and, with JM and JFC, critically revised it for important intellectual content. All authors have read and critically revised the manuscript and approved the final version of the manuscript.

## Pre-publication history

The pre-publication history for this paper can be accessed here:

http://www.biomedcentral.com/1471-2474/15/49/prepub

## Access to data

Data generated in the trial belong to Copenhagen University Hospital, Glostrup.
